# Radiation induced temporal lobe necrosis in patients with nasopharyngeal carcinoma: a review of new avenues in its management

**DOI:** 10.1186/1748-717X-6-128

**Published:** 2011-09-30

**Authors:** Jing Chen, Meera Dassarath, Zhongyuan Yin, Hongli Liu, Kunyu Yang, Gang Wu

**Affiliations:** 1 Cancer Centre, Union Hospital, Tongji Medical College, Huazhong University of Science and Technology, Wuhan, 430022, China; 2 Department of Oncology, Queen Victoria Hospital, Candos, Quatre-Bornes, Mauritius

**Keywords:** Nasopharyngeal cancer, radiation therapy, temporal lobe necrosis, Bevacizumab

## Abstract

Temporal lobe necrosis (TLN) is the most debilitating late-stage complication after radiation therapy in patients with nasopharyngeal cancer (NPC). The bilateral temporal lobes are inevitably encompassed in the radiation field and are thus prone to radiation induced necrosis. The wide use of 3D conformal and intensity-modulated radiation therapy (IMRT) in the treatment of NPC has led to a dwindling incidence of TLN. Yet, it still holds great significance due to its incapacitating feature and the difficulties faced clinically and radiologically in distinguishing it from a malignancy. In this review, we highlight the evolution of different imaging modalities and therapeutic options. FDG PET, SPECT and Magnetic Spectroscopy are among the latest imaging tools that have been considered. In terms of treatment, Bevacizumab remains the latest promising breakthrough due to its ability to reverse the pathogenesis unlike conventional treatment options including large doses of steroids, anticoagulants, vitamins, hyperbaric oxygen and surgery.

## Introduction

Nasopharyngeal cancer (NPC) is highly prevalent in Southern China, particularly in Guangdong province and in the northern parts of Africa and Inuits of Alaska [1]. Till date radiotherapy remains the mainstay treatment of NPC [2]. A definitive radiation dose between 66 Gy and 70 Gy needs to be given to the gross tumor volume (GTV), and 54-60 Gy to the clinical target volume (CTV). More than 70% of patients with NPC present with stage III or IV disease, among whom extensive skull base invasion or even cavernous sinus involvement commonly occur [3]. Treatment with radiation therapy under these circumstances exposes parts of the temporal lobes to doses over 60 Gy. This greatly increases the risks of temporal lobe necrosis (TLN) which is one of the most debilitating late stage complications after radiotherapy in NPC.

The majority of radiation induced TLN patients with NPC that have been reported in the literature were treated with conventional 2D radiotherapy rather than 3D or IMRT. An incidence of TLN of 4.6% in 10 years (conventional fractionation) [4] to 35% in 3.5 years (accelerated fractionation to 71.2 Gy) [5] has been observed. Classical histological findings of TLN include various degrees of coagulative necrosis of brain parenchyma associated with fibrinoid changes of blood vessels while demyelination without blood vessel changes may be observed in less severely affected areas [6]. Other histological features include oligodendrocyte dropping out, axonal swelling, reactive gliosis, and disruption of the blood brain barrier [7,8].

Clinical presentations of TLN are variable, and four main types have been well described by Lee et al [9]. 39% of their patients presented with vague symptoms including occasional dizziness and impairment of memory and personality changes, 31% had features of temporal lobe epilepsy, 16% had no signs or symptoms and were incidentally diagnosed during investigation for other neurologic and endocrine dysfunction after radiation therapy, while 14% of the patients suffered from symptoms of raised intracranial pressure and nonspecific symptoms like mild headache, mental confusion and generalized convulsion as a result of mass effect.

Differential diagnosis of TLN includes intracranial extension of NPC, second primary intracranial malignancies, hematogenous cerebral metastasis and brain abscess [10]. It is easier to exclude brain abscess on the basis of symptoms and laboratory investigations suggestive of infection. On the other hand, hematogenous cerebral metastasis from NPC are extremely rare [11].

Both tumor and radiation necrosis can cause vasogenic edema, disrupt the blood-brain barrier and cause cavitations. Clinically, both conditions can present with features of raised intracranial pressure and show contrast enhancement on MRI. Thus, a diagnostic dilemma sometimes arises when trying to differentiate TLN from neoplasm (intracranial extension of NPC or a second primary intracranial malignancy). We recently reported a case report about such an ambiguous situation leading to delay in the institution of the appropriate treatment [12]. Many times a working diagnosis can still be reached without resorting to biopsy by carefully correlating the history, reviewing the treatment plan, correlating the high dose volume with TLN and the findings on conventional imaging. Yet, the lack of specificity of conventional imaging has prompted the search for a more reliable diagnostic tool.

## Diagnostic Modalities

### Conventional imaging

Among the different anatomical imaging available, MRI appears to have higher sensitivity than CT in diagnosing TLN. However, CT scan is best suited to rule out skull base erosions [13]. Warranting brief attention are two characteristic features of TLN on CT: the early finger like hypodense area representative of reactive white matter edema and the late cyst like changes corroborating with liquefactive necrosis and surrounding gliosis [9]. The finger and the cyst signs on CT are seen as irregular and rounded lesions on MRI respectively [13]. The features in favor of TLN include two characteristic enhancement patterns - the "Swiss cheese" and "soap bubble" [14,15]. Also, TLN lesions are usually restricted within the portals of radiation though they may extend well beyond.

## Advanced Imaging Tools

Advanced imaging techniques are mainly functional imaging techniques which assess physiological parameters and can provide additional information about the lesions.

### Perfusion and diffusion weighted MRI

Perfusion MRI allows a non-invasive evaluation of cerebral blood flow (CBF) and relative regional cerebral blood volume (rrCBV). Neovascularised tumors manifest a higher CBF due to the high blood volume and blood flow to the tumor bed. On the other hand, temporal lobe radiation necrosis exhibits low vascularity and hence a lower CBF. Dynamic susceptibility contrast MRI is a form of perfusion MRI, during which dynamic MRI images is rapidly taken over time, after the patient is given a bolus injection of a paramagnetic contrast medium. During the perfusion phase, the contrast enters the intravascular compartment and is recorded as a drop of signal intensity. However, as the contrast medium moves rapidly into the extracellular compartment at the end of the perfusion phase, a rise in signal intensity is noted. The transient drop in the signal intensity is prominent in tumors, as a result of their increased angiogenesis, that results in the magnetic susceptibility effects of contrast accumulation in the intravascular compartment. Several parameters including cerebral blood flow, time to enhancement, and cerebral blood volume can be evaluated from this technique. Tsui et al used dynamic susceptibility contrast MRI to study the rrCBV of nine NPC after radiotherapy who presented with clinical features of temporal lobe necrosis [16]. In this study, all but one patient had low signal on T1 and high signal on T2 images with heterogeneous enhancement and demonstrated marked hypoperfusion on the rrCBV maps. A recent study suggests that perfusion MRI might be superior to FDG PET and C-MET [17]. It also offers the advantage that it can be performed at the same time as conventional MRI. The potential pitfalls of perfusion MRI include susceptibility artifacts, relative but not absolute quantification of CBV and inaccurate determination of CBV in cases of severe disruption or absence of blood brain barrier [18].

Perfusion MRI can be used in conjunction with diffusion weighted MRI. It is speculated that a failure of the Na^+^-K^+ ^pump leads to an influx of water from the extracellular compartment to intracellular space which forms the basis of the net decrease of diffusion coefficient [19]. In the brain parenchyma the diffusion of water is impeded by various structures including membranes and myelin sheath so that presence of tumor further impedes water movement due to the added cell membrane mass. Apparent diffusion coefficient (ADC) maps are obtained which may be compared with rCBV maps of perfusion MRI for 'mismatch'. Radiation necrosis generally displays marked high diffusion on ADC while the relative CBV map reveals marked hypoperfusion due to damage of the endothelial cells and ischemia leading to a "diffusion and perfusion mismatch" [20]. Tsui et al established the diagnosis of temporal lobe necrosis of 16 NPC patients who developed clinical symptoms or ambiguous radiation induced temporal lobe abnormalities on conventional MRI by diffusion and perfusion MRI [21]. He noted a larger abnormality on the rCBV map compared to the ADC map which he concluded was due to presence of injured but potentially salvageable brain tissue. However, paradoxical findings have also been obtained with this technique in patients presumably with radiation induced brain necrosis. Le Bihan et al reported a low ADC value in radiation necrosis patients [22]. This may be due to the fact that radiation induced necrosis is usually composed of a mixture of different components. Further prospective studies are required to clearly establish the clinical usefulness of the mismatch pattern.

### Magnetic resonance spectroscopy

Whereas MRI provides morphological information, MR spectroscopy allows direct, noninvasive quantification of various metabolites and the study of their distribution in different tissues. Metabolites, such as choline (Cho), N acetyl aspartate (NAA), creatinine (Cr) and lipid-lactate (Lip-Lac) spectrum, are quantified. Lip-Lac peaks reflect anaerobic metabolism. Increased choline levels represent enhanced cellular membrane phospholipid synthesis accompanying tumor cell proliferation [23,24]. Areas believed to be radionecrotic will usually show lowered Cho while high Cho is obtained in areas with dense viable tumor cells. NAA functions as a neuronal integrity marker and is decreased in both tumor and radionecrosis due to neuronal destruction. A decrease in NAA levels on single voxel MR spectroscopy was reported in all 18 NPC patients in a study with imaging evidence of radiation induced TLN, and this decrease was evident even before a change in Cho or Cr levels [25]. Creatinine indicates cellular energy metabolism and is fairly stable under most conditions. It is therefore used as the denominator in metabolic ratio calculations such as Cho/Cr and NAA/Cr ratios, even though some reports have questioned the stability of Cr in tumors, hypoxia and other confounding conditions [26]. MR spectroscopy has been used to differentiate between tumor and radiation changes, and even guide the management of patients as reported by Smith et al [27]. Patients with a Cho/NAA ratio of less than 1.1 were assigned for imaging follow-up; those with a higher ratio of more than 2.3 underwent immediate treatment in line with tumor while patients with Cho/NAA ratios between these values would undergo biopsy. As a drawback, MRS lacks the ability to precisely identify the boundaries of a tumor and radiation necrosis when they co-exist at the same location. There is no consensus yet on the calculated threshold which can best distinguish radiation necrosis from a tumor. Unlike PET, MRS does not have the disadvantage of ionizing radiation. However, MRS and PET still play a complementary role in classifying indeterminate brain lesions into non-neoplastic and neoplastic.

### Positron emission tomography

The use of functional FDG PET appeared to be promising on a theoretical basis by measuring the uptake of 2-[¹⁸F] DeoxyGlucose (FDG). Tumors are thought to be usually hypermetabolic and thus show an increased uptake of FDG, while radiation necrosis is hypometabolic. Di Chiro et al reported a 100% sensitivity and specificity with PET in the differentiation of tumor from radiation necrosis in one of the largest samples of patients where all cases were pathologically confirmed [28]. Studies carried out after 1990s, unfortunately have defied the above conclusion [29-33]. PET has been shown to have a high sensitivity of about 80% but low specificity of 40%. Causes of false negative PET scanning of a tumor include recent radiation therapy, low histological grade and small tumor volume, while false positive PET in radiation-induced brain injury could be due to activated repair mechanisms or inflammatory activity [34]. It is therefore suggested that GdTPA MRI should be used in conjunction with FDG PET when making a diagnosis of a suspected case of radiation necrosis [34]. PET also has the disadvantage of being expensive, not widely available and exposing the patient to radiation.

In order to improve its specificity, different radiopharmaceuticals have been tried like the ^13^N-NH3 [35] and ^11^C Methionine (MET) in place of FDG. 11 C-methionine is the commonest amino acid tracers that has been studied. Methionine, is one of the essential amino acids which is required for protein synthesis and its derivatives S-adenosyl methionine acts as a methyl donor as well as a precursor for the synthesis of polyamine. Due to an increase in these activities, in cases of malignancy, an increase uptake of this tracer is observed in such patients [36]. Since the uptake of amino acid is low in normal brain tissue as compared to tumor, a better contrast can be obtained between the two, with MET-PET scanning as opposed to FDG-PET [37]. MET-PET allows for the identification of low grade brain tumors including gliomas, even when no uptake is visible on FDG PET [37]. The high cost and limited availability of PET scans spurred the consideration of alternative imaging tools such as Thallium-201 single photon emission computed tomography (^201^Tl SPECT).

### Single photon emission computed tomography

^201^Tl SPECT is efficacious and a less costly method compared to PET. Thallium is a potassium analog that has been used for many years in myocardial perfusion imaging. It is presumed that the uptake of Thallium by tumor cells relies on a combination of mechanisms including blood brain barrier disruption, blood flow and Na^+^/K^+ ^ATPase pump activity [38,39]. It can differentiate between tumor and radiation necrosis and even estimate the grade of a tumor [38]. It reflects viable tumor burden more accurately than CT, MR, or other radionuclide studies [40-43]. Radiation induced necrotic tissue does not take up Thallium-201 due to lack of the active transport mechanism and Na^+^/K^+ ^ATPase enzyme while tumor cells have increased levels of this enzyme, therefore concentrate Thallium-201. Moreover, Thallium is taken up in increasing amounts with increasing histological grade of the tumor. The slightly lower spatial resolution compared to PET is one of the main setbacks of SPECT.

## Other Clues For Diagnosis

The levels of circulating plasma EBV DNA levels may contribute in differentiating between tumor and TLN. Measurement of free plasma EBV DNA has been found to be a highly specific and sensitive marker of nasopharyngeal carcinoma [44]. EBV DNA is released into blood after lysis of NPC cells and hence reflects the tumor load. Hou et al found that pre-treatment plasma EBV DNA concentrations significantly correlated with tumor volume, T stage and TNM stage. They also believe that pre-treatment EBV DNA concentrations mainly reflect tumor load whereas post treatment EBV DNA concentrations are an important predictive factor for distant metastases [45]. Leung et al reported that pre-treatment plasma EBV DNA concentrations could predict distant metastasis in early stage NPC [46]. Lo et al also showed that circulating plasma EBV DNA copies increase significantly in NPC patients with tumor recurrence [44], and the EBV DNA levels can significantly increase sometimes up to 6 months earlier than clinical diagnosis. A considerably high pretreatment level of EBV DNA and a subsequent rise during follow up may therefore indicate tumor recurrence and may aid in differentiating tumor from TLN in ambiguous circumstances.

Despite the multiple attempts to distinguish tumor from TLN by radiological methods, biopsy still remains the most reliable way to reach an unequivocal diagnosis since no radiological technique has yet the capacity to reliably differentiate between these two entities.

## Prevention

Prevention remains the cornerstone of a successful therapeutic algorithm for TLN. It is practically impossible to completely shield the temporal lobes during radiotherapy for NPC patients with skull base invasion or cavernous sinus involvement. Kam et al showed that IMRT significantly limits the maximal dose to the temporal lobes to 46 Gy as compared to 66.5 Gy in 2D radiotherapy in NPC patients with T4N2M0 disease [47]. Additionally, replanning for patients with NPC before the 25th fraction during IMRT further helps to ensure adequate dose to the target volumes and safe doses to critical normal structures, which may decrease incidence of TLN [48,49].

Among the multiple etiologies of TLN, the fraction size is of utmost significance [4]. Lee et al analyzed the incidence rate of temporal lobe necrosis in 1008 patients treated radically with different fractionation schedules for T1 NPC [4]. 621 patients, who received a lower total dose of 50.4 Gy in 4.2 Gy per fraction, had a significantly higher 10 year actuarial incidence rate of TLN compared to the 320 patients who received a higher total dose of 60 Gy but in 2.5 Gy per fraction: 18% versus 4.6% respectively. Apart from fractional dose, in a later study, they identified the overall treatment time and the twice daily schedule as additional etiologic factors of TLN [50]. The 5 year actuarial incidence of TLN in this study ranged from 0% in patients who had received 66 Gy in 2 Gy per fraction once daily as compared to 14% in patients who received two fractions per day during part of the treatment (71.2 Gy in 40 fractions in 35 days). Furthermore, in a retrospective analysis of 849 NPC patients treated with radiation therapy alone, Yeh et al observed that patients receiving external beam radiation dose more than 72 Gy experienced a higher incidence of temporal lobe necrosis [51]. Moreover, the use of boost has improved local control, especially of locally advanced NPC but at the cost of an increase in toxicity. Hara et al reported a 12% incidence of TLN among their 82 NPC patients at a median follow up of 3.4 years, who were treated with external beam RT to 66 Gy followed by sterotactic radiotherapy (SRT) boost of 7-15 Gy in a single fraction [52]. Likewise, during a 5 year follow-up, Lee et al observed a rate of 8.3% of TLN among 33 patients of theirs, who received 5 Gy SRT boost in 2 fractions after conformal RT to a total dose of 70 Gy [53].

By stringently limiting doses to the temporal lobes, using conventional fraction size, adoption of IMRT and replanning during IMRT, occurrence of TLN can be prevented in most patients. Control of comorbid factors like hypertension, diabetes, lipidemia, obesity and smoking, which are known contributory factors in the development of TLN, may also reduce the incidence and severity of the sequelae.

## Treatment

Treatment of TLN is still a challenging issue. Treatment modalities for cerebral radio-necrosis are also suitable for radiation-induced TLN. Observation may be the only treatment needed in some selective patients with TLN including those that are asymptomatic, have a long latency period of TLN development, have received only one course of radiotherapy and have favorable MRI findings [54].

### Steroids

Steroids have been used to provide prompt symptomatic relief. Radionecrosis is usually associated with various degrees of white matter edema in the early phase, which acts as a space occupying lesion and hinders the blood supply to the temporal lobe. Steroids help decrease cytokines and inflammatory reaction which not only decreases cerebral edema but also minimizes the risk of subsequent development of vascular and inflammatory changes [55]. Unfortunately, they are seen to be beneficial only in early phase of extensive liquefactive necrosis[9]. Tapered doses of dexamethasone achieved a durable response in 25 out of 72 NPC patients studied by Lee et al who had radiation induced TLN [9]. The reported doses of dexamethasone used range from 4-16 mg/day for 4-6 weeks and were gradually tapered off [9]. Prolonged use of steroids is associated with various side effects like diabetes, myopathy and weight gain [56]. However, steroids also have immunosuppressive effects on the already immuno-compromised cancer patients and put them at high risk of developing fatal sepsis and death [9]. Furthermore, since steroids do not reverse the pathogenesis, many patients experience the symptoms again after they are tapered off the medication [7]. In a recent study, pulsed steroid treatment, which has better tolerance and may minimize long term steroid induced side effects, has been compared to oral steroids in the treatment of TLN in NPC patients. The clinical and radiological outcomes were found to be better with the use of pulsed steroids. 20% patients experienced radiological improvement as opposed to 3.2% of patients receiving conventional oral steroids (p < 0.0001) which could be due to the comparatively lower pulsed-steroid dose used as compared to oral steroids. These results should be interpreted with caution as baseline characteristics and the follow-up protocols of the treatment groups were different [54].

### Anticoagulants, anti-platelets and vitamins

Glantz et al were the first to report the use of heparin followed by warfarin for 3-6 months in an attempt to treat radiation necrosis by arrest and reversal of endothelial injury which is the predisposing lesion entailing to radiation necrosis [57]. This therapeutic option was met with little success since the symptoms reemerged after their discontinuation. Anti-platelet treatment with pentoxyfyllin, aspirin, and ticlopidine have also been used to prevent thrombosis of the blood vessels but the potential risk of bleeding from these agents should be considered [7]. At present, there are still no large clinical trials to support their routine use in the treatment of radiation induced necrosis. High doses of vitamins such as alpha tocopherol has shown the ability to improve the neurocognitive function of radiation induced temporal lobe necrosis in NPC patients in a phase II trial when it was administered for a period of 1 year [58].

### Hyperbaric oxygen

Hyperbaric oxygen (HBO) has also been tried in radiation necrosis patients [59]. It raises the Pa0_2 _of tissues and initiates cellular and vascular repair. Oxygen is delivered at 2.0 to 2.4 atm in 20-30 sessions for 90-120 min per session. Chuba et al treated 10 patients of radiation necrosis with hyperbaric oxygen among whom 6 showed improvements with 3 having documented radiographic response [60]. However, many also received concomitant steroid treatment. Serious complications of hyperbaric oxygen are rare but may include oxygen toxicity and closed cavity barometric pressure trauma. There have been concerns about tumor regrowth with increased oxygenation, but this has not been supported by Feldmeier [61,62]. However, a recent study suggests that HBO therapy may increase risk of cancer re-recurrence in patients who had locoregional recurrence and successfully salvaged head and neck cancer [63]. Further studies are required to confidently establish its efficacy and safety and understand its implication in treatment of TLN.

### Surgery

Surgery is usually reserved as the last resort in patients with significant increase in intracranial pressure or in those with progressive neurological deficits despite steroids or other medical therapy [31]. It may also be indicated in cases of TLN complicated by hemorrhage or brain abscess formation [8,64]. Previously, conflicting outcomes have been obtained with neurosurgery, with good outcomes in some [65] while poor in others [9]. Recently, Mou et al performed surgery for 14 patients with histologically confirmed TLN, who failed to show improvement with steroids [66]. Good surgical outcome with significant symptom improvement and low recurrence rate was obtained. The results from the above study depict that surgery may not only cause partial reversal of the radionecrotic process, but also halt the progression of radiation necrosis. Similar results were reported in an another recent study where 27 radiation induced TLN patients with NPC had emergency life saving neurosurgery [54]. Generally, patients with good performance status, well-controlled primary disease and good prognosis may be expected to fare better with surgery. Until now surgery has only been performed in a small sample size and many questions concerning its use still warrant further clarification.

### Bevacizumab

Bevacizumab is the latest addition to the therapeutic options for radiation induced TLN. Gonzalez et al firstly reported a group of 8 patients with radiation-induced brain necrosis treated with bevacizumab on either a 5 mg/kg/2-week or a 7.5 mg/kg/3-week schedule. In all 8 patients significant reductions in dexamethasone dose as well as abnormalities on MRI fluid-attenuated inversion-recovery (FLAIR) corresponding to edema and T1-weighted post-Gd-contrast abnormalities corroborating with capillary permeability were noted [67]. Similar observations were reported in other studies [68]. These remarkable changes are thought to be the result of normalization of the blood brain barrier by bevacizumab [67]. Besides improvement in imaging parameters, Wong et al reported significant improvement of neurocognitive deficits [69]. It is postulated that fibrinoid necrosis of blood vessels and hypoxia leads to VEGF release [70]. Additionally, radiation-induced damage of astrocytes further causes leakage of VEGF. This then acts on the capillary targets and causes neovascularization. The new vessels are leaky and further perpetuate edema and blood brain barrier disruption [70,71]. Bevacizumab, therefore seems to have both a diagnostic and therapeutic role. A randomized double-blind placebo-controlled trial of bevacizumab therapy for radiation necrosis of central nervous system, involving 14 patients, was carried out [72]. A dose of 7.5 mg/kg of bevacizumab was administered 3 weekly in one group while the other group received intravenous placebo [72]. Final results depicted that all bevacizumab-treated patients, while none of the placebo-treated patients showed improvement in neurological symptoms or signs. However, one of the limitations of this study remains its small sample size.

Preliminary results have shown that bevacizumab at a dose of 7.5 mg/kg every 3 weeks, or 5 mg/kg every two weeks for 12 weeks can stop the progression of radiation necrosis, or even reverse its process in most patients with limited follow-up [72]. A recent case report has shown a more rapid and early onset of relief of symptoms accompanied with a long lasting response with bevacizumab than steroids in the treatment of TLN in a NPC patient [56]. However, most studies using bevacizumab to treat brain necrosis involve small sample size, and their longest follow-up is just 10 months. It is hence necessary to prolong the follow-up to see whether the efficacy is durable or not. For example, a recent case report from Japan described two patients with radiation necrosis treated with 5 mg/kg of bevacizumab biweekly for 6 cycles [73]. There was an improvement in neurocognitive function and perifocal edema. However, signs of radiation necrosis appeared again several months after discontinuation of the drug. Fortunately the patients still responded to a second course of bevacizumab. This case report as well as previous studies emphasize on the need for further research to determine the optimal dose, the longest interval between doses to achieve durable resolution of CNS radiation necrosis and its long term safety through larger sample size studies.

More recently, the use of mouse nerve growth factor for 2 months in a NPC patient with clinical and radiological manifestations of radiation induced TLN showed complete resolution of the MRI abnormalities and an improved cognitive function in one case report [74]. However, no definite conclusions can be formulated from this report since it involves only one patient whose TLN was not confirmed by histology and the follow up period is short. Hence further studies are required.

## Conclusion and Perspectives

After a brief analysis of all the evidence available until now concerning the diagnosis and treatment of TLN, we still do not have a definite algorithmic management for this entity. However, a few conclusions can still be drawn:

**1. **Prevention is always better than treatment in the management of TLN. IMRT is definitely the radiotherapeutic technique of choice for treatment of NPC at present, considering its normal organ sparing ability together with its capacity to achieve adequate tumoricidal dose. Furthermore, the mutifactorial etiologies of TLN including dose fraction size should always be kept in mind when treating NPC patients.

**2. **In cases where a definite diagnosis of TLN is difficult to make according to conventional radiological modalities like CT and MRI, a panoply of functional imaging techniques including MRS, perfusion MRI, and PET might aid in diagnosis. Furthermore, evaluation of EBV DNA plasma level can provide a clue.

**3. **Until now conventional treatment including steroids and anticoagulants fails to reverse the pathogenesis of TLN and merely used for palliation. However, recently bevacizumab has gained much interest in the management of this entity since it holds the potential of reversing the underlying pathogenesis. Since its use is still in the initial phase, critical questions such as its optimal dose and duration of administration still needs further investigation.

**4. **Further elucidation of the pathogenesis of TLN at the molecular level may open new frontiers in therapeutic options.

## Competing interests

The authors declare that they have no competing interests.

## Authors' contributions

All authors read and approved the final manuscript. JC and MD drafted the manuscript together. ZY and HL carried out data collection. GW gave a lot of instructions in writing the review. KY took charge of the whole work.
